# Thallium Removal from Aqueous Solutions Using L Zeolite: Structural Modifications, Cation Distribution and Water Network Reorganisation

**DOI:** 10.3390/molecules31122130

**Published:** 2026-06-17

**Authors:** Luca Adami, Maura Mancinelli, Francesco Di Benedetto, Renzo Tassinari, Matteo Alberghini, Giacomo Ferretti, Annalisa Martucci

**Affiliations:** 1 Department of Physics and Earth Sciences, University of Ferrara, Via Saragat 1, 44122 Ferrara, Italy; maura.mancinelli@unife.it (M.M.); francesco.dibenedetto@unife.it (F.D.B.); tsr@unife.it (R.T.); 2 Department of Environmental and Prevention Sciences, University of Ferrara, Via Borsari 46, 44121 Ferrara, Italy; lbrmtt1@unife.it; 3 Department of Chemical, Pharmaceutical and Agricultural Sciences, University of Ferrara, Via Borsari 46, 44121 Ferrara, Italy; frrgcm@unife.it

**Keywords:** thallium removal, K-L zeolite, ion exchange, structural modifications, water network reorganisation, adsorption behaviour, hydration network

## Abstract

This study investigates potassium-L zeolite (K-L) as an adsorbent for the removal of thallium (Tl^+^) from aqueous solutions, focusing on the relationship between cation exchange and framework structural response. X-ray powder diffraction (XRPD), thermal analysis, and Rietveld refinements were employed to monitor structural modifications upon Tl^+^ uptake, combined with batch adsorption experiments to evaluate the removal performance. At low Tl^+^ uptake, only minor structural perturbations occur, mainly involving slight shifts in extra-framework cation positions and limited rearrangement of channel water molecules. At higher Tl^+^ concentrations, a measurable anisotropic expansion of the zeolite framework is observed, consistent with partial substitution of K^+^ by Tl^+^ and progressive modification of the hydration environment within the pores. Moreover, the crystallographic distribution of Tl^+^ differs from that of the original K^+^ cations, suggesting a specific site preference during the uptake process. Batch experiments reveal rapid uptake kinetics, with equilibrium reached within minutes, and high removal efficiency up to 99.5%. The adsorption behaviour is well described by the Langmuir model, with a maximum adsorption capacity of 631 mg g^−1^. These findings highlight the coupling between ion exchange and structural flexibility in K-L zeolite and support its potential application for efficient thallium removal from contaminated water.

## 1. Introduction

Thallium (Tl) is a heavy, soft, and malleable metal naturally present in trace amounts, with toxicity comparable to that of mercury, cadmium, and lead [1,2]. In aqueous systems, it occurs mainly as Tl^+^, generally the most stable and mobile oxidation state, while Tl^3+^ can persist under specific photochemical or microbial conditions [3]. Tl^+^ predominates as the free cation Tl^+^ over a wide pH range (4–9), whereas Tl^3+^ forms stable hydroxylated complexes. Microbial transformation may produce dimethyltallium ((CH_3_)_2_Tl^+^), an organic species that can account for up to 48% of total thallium in some deep-ocean samples [3]. Tl concentrations in the Earth’s crust range from 0.1 to 1.7 mg/kg, commonly associated with Zn, Cu, and Pb sulfide minerals and coal, with higher levels in granites, organic-rich shales, and Jurassic coals [2]. Local geogenic enrichment can result in elevated soil concentrations, such as 3.7 mg/kg at the Allchar mining site (former Yugoslavia) due to historical antimony extraction, where arsenic and Tl occur as impurities [2,4]. Anthropogenic activities—including fossil fuel combustion, non-ferrous metallurgy, and mining—further contribute to Tl contamination. The metal accumulates in clay- and organic-rich soils containing Fe and Mn oxides, with mobility enhanced under acidic conditions [5]. Groundwater affected by pyrite-rich deposits or mining residues may reach concentrations far above regulatory limits, for example 1000–9000 μg/L in Tuscany, 1440 μg/L in Guizhou (China), and 5–99 μg/L in Germany [1,6,7,8,9,10], highlighting both natural and anthropogenic sources of risk.

Biomonitoring studies confirm that chronic Tl exposure occurs even at low environmental concentrations, affecting humans and wildlife. Tl accumulates in invertebrates and fish (20–75%), mainly in the cytosol [11], disrupting metabolic enzymes such as glutathione S-transferase (GST) and nucleoside diphosphate kinase (NDPK), as well as the glutathione-based antioxidant system [3]. In humans, exposure via drinking water, food, and inhalation induces oxidative stress, K^+^ channel disruption, interactions with thiol-containing proteins, apoptosis, endoplasmic reticulum stress, and genotoxicity [1,3,6,11]. Chronic low-dose exposure can cause non-specific symptoms and poses potential risks to renal function and foetal development [4,5]. The high toxicity, mobility, and environmental persistence of Tl make its removal from contaminated waters a relevant problem in both environmental geochemistry and water-treatment technology. Several treatment strategies have been proposed for Tl removal from water and wastewater. Oxidative approaches, often involving Mn-, Fe-, Al-, or Ti-based reactive materials, are relevant because Tl^+^ can be oxidised to less mobile Tl ^3+^ species, followed by adsorption or precipitation [11,12,13]. Regulatory approaches such as the US EPA Best Demonstrated Available Technology (BDAT) are based on Tl^+^ oxidation followed by precipitation to achieve residual concentrations <140 μg/L [11,12,13]. Other limits include 2 μg/L in drinking water, 4 μg/L in seawater and 1 mg/kg in soils [13]. However, adsorption- and ion-exchange-based processes remain particularly attractive because they can be implemented under mild conditions and may allow selective capture of Tl^+^ without extensive chemical conditioning of the water matrix.

Recent studies have investigated several classes of adsorbents for Tl removal, including γ-alumina nanoparticles, manganese oxides, magnetite-based materials, titania, Prussian blue analogues, macrocyclic compounds, activated carbon, industrial residues, biomass-derived sorbents, and natural minerals such as illite [14,15,16,17]. Although several of these materials show high Tl^+^ affinity, their practical application may be limited by selectivity, regeneration, secondary release, nanoparticle recovery, or long-term stability. Zeolites represent a distinct class of sorbents because their ion-exchange properties are directly linked to crystallographically defined extra-framework cation sites, allowing macroscopic removal performance to be correlated with atomic-scale structural information. This aspect is particularly relevant for Tl^+^, whose monovalent charge and ionic size make it suitable for exchange with alkali cations hosted in aluminosilicate frameworks.

Among zeolitic materials, potassium-form zeolite L, K-LTL is of specific interest because its one-dimensional channel system contains exchangeable K^+^ ions located at crystallographically distinct sites. To place the performance of K-LTL in the context of existing Tl sorbents, Table 1 compares representative materials reported in the literature in terms of maximum adsorption capacity, dominant removal mechanism, and regeneration or stability limitations. This comparison is not intended to rank sorbents solely by capacity, since uptake values depend strongly on experimental conditions, but rather to identify the structural and mechanistic advantages of a crystalline ion-exchange host whose Tl^+^ uptake can be investigated by diffraction methods.

**Figure 1:**
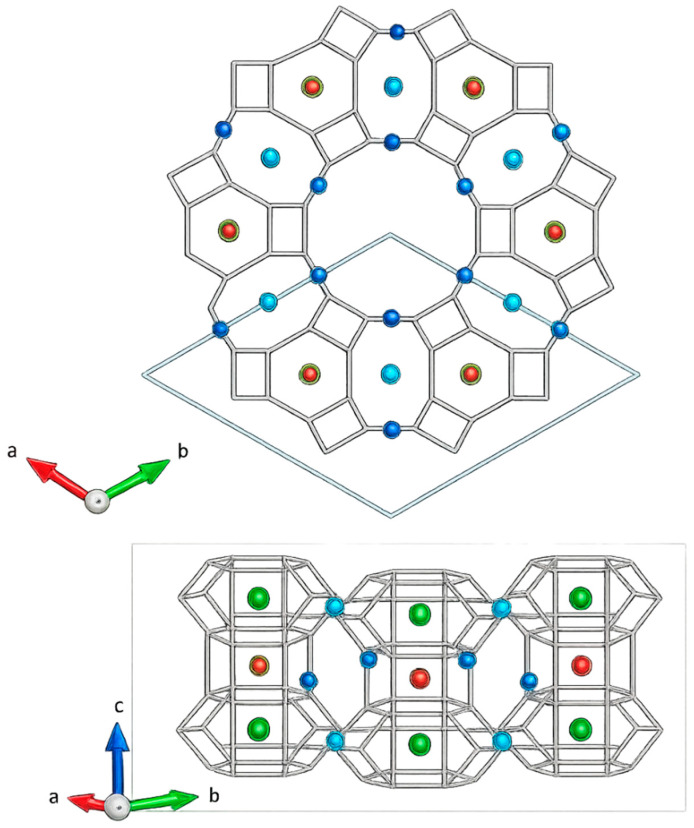
Cation sites KB, KC, KD and K4 in the LTL framework. (Top panel): The hexagonal unit-cell is shown in white with a view down the 12R channel running parallel to the crystallographic *c*-axis. (Bottom panel): Cationic sites are located in the stacks of *can* cages and d6r units surrounding the 12R channels; site KB (z = 0) is at the centre of the *d6rs* (red), site KC (z = 0.5) is at the centre of the *can* cages (green) and sites KD and K4 (z = 0) in the windows of the elliptical 8R channels (blue and light blue).

The high Tl^+^ uptake capacity of K-LTL (631 mg g^−1^) listed in Table 1 is related to its specific crystallographic architecture and to the accessibility of exchangeable K^+^ sites within the LTL channel system. Zeolite L is a large-pore material with ideal composition K_6_Na_3_Al_9_Si_27_O_72_·21H_2_O, LTL-framework type, space group *P*6/*mmm* and unit cell parameters a = 18.40(4) Å and c = 7.52(3) [27]. The first refined structural model of synthetic zeolite L was provided by Barrer and Villiger [28] using X-ray powder diffraction. The framework consists of large one-dimensional channels oriented along the *c*-axis and delimited by 12-membered tetrahedral rings (12MRs), together with narrower elliptical channels delineated by 8-membered rings (8MRs). The 12MR channels are flanked by vertical columns composed of alternating cancrinite cages (*cans*) and double six-ring units (*d6rs*), with elliptical 8MR channels located between these columns (Figure 1). The inner diameters of the channels range from approximately 7.1 Å to 12.6 Å, and the centre-to-centre distance between adjacent channels is 18.4 Å [27]. The framework contains two distinct tetrahedral sites, T1 and T2, with Al preferentially occupying T1 according to neutron diffraction studies [29].

In the as-synthesised form, K^+^ ions occupy several extra-framework sites commonly designated as A–D [30,31,32]. Site A is located at the centre of the *d6r* units (*z* = 0), site B at the centre of the *can* cages (*z* = 1/2), site C within the window between the 8MR channel and the wall of the 12MR channel (*z* = 1/2), and site D at the periphery of the 12MR channel (*z* = 0). Previous structural studies indicate that exchange preferentially involves the more accessible sites within or near the main channels, particularly sites D and C, followed by less accessible positions [28,29,30,31,32]. This crystallographic distribution of K^+^ sites provides the structural basis for investigating whether Tl^+^ uptake occurs through selective K^+^/Tl^+^ exchange, site redistribution, or additional framework relaxation [28,29,30].

The LTL framework is known to accommodate different mono-, multi-, and rare-earth cations, including Cs^+^, Li^+^, and Ce^3+^, through topology-dependent structural adjustments involving changes in channel occupancy, pore geometry, and extra-framework cation distribution [17,30]. These properties have supported the use of LTL-type zeolites in catalysis, optical materials, sensors, and electronic applications [33,34,35,36]. However, despite the environmental relevance of Tl^+^ and its chemical affinity for K^+^-bearing phases, detailed crystallographic information on Tl^+^ incorporation into K-LTL under hydrated ion-exchange conditions remains limited. Previous studies on Tl^+^ removal by zeolites have mainly focused on adsorption capacities, selectivity, and exchange performance, whereas structural studies of LTL-type zeolites have largely addressed the incorporation of alkali, alkaline-earth, and rare-earth cations. In particular, the relationship between Tl^+^ uptake, K^+^/Tl^+^ exchange, cation-site redistribution, framework deformation, and hydration changes have not been systematically resolved for this zeolite [17,30,31,32,33,34,35,36].

From a stereochemical perspective, Tl^+^ incorporation into K-LTL is favoured by the monovalent character of both Tl^+^ and K^+^ and by their comparable ionic radii. Nevertheless, Tl^+^ cannot necessarily be treated as a purely electrostatic extra-framework cation. Structural studies on Tl-bearing aluminosilicates, including Tl-containing sodalite-type phases [37,38], indicate that Tl–O interactions may display partial covalent character, reflected by specific Tl–O bond distances and local structural distortions. Although sodalite and LTL differ in framework topology, pore connectivity, and cation-site geometry, these observations suggest that Tl^+^ retention in K-LTL should be evaluated at the crystallographic level rather than inferred solely from adsorption capacity or solution chemistry.

Accordingly, the novelty of the present work is the direct correlation of Tl^+^ removal by K-LTL with the crystallographic response of the zeolite framework under hydrated exchange conditions. The study combines adsorption experiments, ICP-MS chemical analysis, XRPD Rietveld refinement, Fourier electron-density mapping, and thermal characterisation to investigate Tl^+^ migration, K^+^/Tl^+^ exchange, extra-framework cation redistribution, framework deformation, and hydration changes within the LTL channels. By resolving the location and local coordination environment of Tl^+^ after uptake, this work addresses whether thallium is only removed from solution by ion exchange or is structurally stabilised within specific extra-framework sites of the LTL host. This structural information provides a basis for assessing the retention mechanism of Tl^+^ in K-LTL and for evaluating its potential as a crystalline ion-exchange material for thallium remediation.

## 2. Results and Discussion

### 2.1. Quantitative and Statistical Analysis of ICP-MS Data

ICP-MS analyses were performed to quantitatively determine thallium concentrations in L-zeolite and to compare them with both the corresponding water analyses in low-concentration samples and the estimates obtained from Rietveld refinement in high-concentration samples. After appropriate sample dissolution and instrument calibration at different times, measurement errors were minimised and the resulting data were found to be consistent and complementary.

The analyses indicate that the pristine L-zeolite, not treated with thallium-containing solutions, shows no detectable traces of this element, with a concentration less than 0.01 ppb.

For the sample treated with low thallium concentration, ICP-MS analyses were performed on the initial thallium-containing aliquot, the post-treatment water, and the post-treatment solid zeolite. The initial thallium concentration in the solution was 510 ppm, whereas the final solution, averaged over three replicates, showed a residual concentration of 10 ppm. This corresponds to a 98% removal and a q_e_ value of 2.48 mg g^−1^ (standard deviation σ = 0.01).

Chemical analysis on the post-treatment solid sample revealed approximately 2155 ppm of thallium. Considering the amount of thallium adsorbed from the solution (500 ppm), the solution volume (1 mL), and the amount of zeolite used (0.2 g), the expected concentration was about 2490 ppm, which is therefore consistent with the experimental value (Table 2). Given that Tl^+^ remains soluble in aqueous systems in the experimentally investigated pH range (3–9.5) [39], precipitation phenomena are unlikely. The small discrepancy between calculated and measured concentrations can therefore be attributed to experimental uncertainties, including minor mechanical losses during handling and dilution steps associated with ICP-MS analysis. Consistently, no additional crystalline phases were detected by XRPD, further excluding the occurrence of bulk precipitation of Tl compounds (Table 2).

Different pH values were also tested to evaluate the zeolite’s removal efficiency under oxidising or reducing conditions. At acidic pH (~3), thallium concentration decreased from 610 ppm to 9 ppm, corresponding to a 98.45% removal efficiency and ~3.00 mg g^−1^ of q_e_ (σ = 0.02). At pH ~ 5.3, the concentration dropped from 630 ppm to 20 ppm, with a removal of 96.88% and ~3.00 mg g^−1^ of q_e_ (σ = 0.09). In basic conditions (pH ~ 9.5), thallium concentration decreased from 620 ppm to 5 ppm, corresponding to a 99.2% removal and ~3.05 mg g^−1^ of q_e_ (σ = 0.01). Although slight variations were observed in thallium residual concentration and removal efficiency under different pH conditions, these differences were not significant (*p* > 0.05). Therefore, neither the removal efficiency (R_e_%) nor the adsorption capacity (q_e_) were significantly affected by the pH value of the solution.

For the high-concentration sample, it was not possible to analyse the post-treatment water. Measurement of the post-treatment solid revealed 346,900 ppm of thallium, indicating material saturation. The initial thallium concentration in the solution was approximately 102,200 ppm and, considering the use of 0.5 g of zeolite in 10 mL of solution, the removal percentage was about 16.9%. From the Rietveld refinement estimate, the theoretical thallium concentration in the sample was 33.6 wt% of the zeolite, corresponding to approximately 336,000 ppm, in good agreement with the experimental value. The slight discrepancy can be attributed to instrumental errors or sample dilution. These data strongly support and confirm the Rietveld refinement estimates (Table 2).

### 2.2. Thallium Uptake, K^+^/Tl^+^ Exchange, and Water-Network Reorganisation in LTL Zeolite

**Figure 2:**
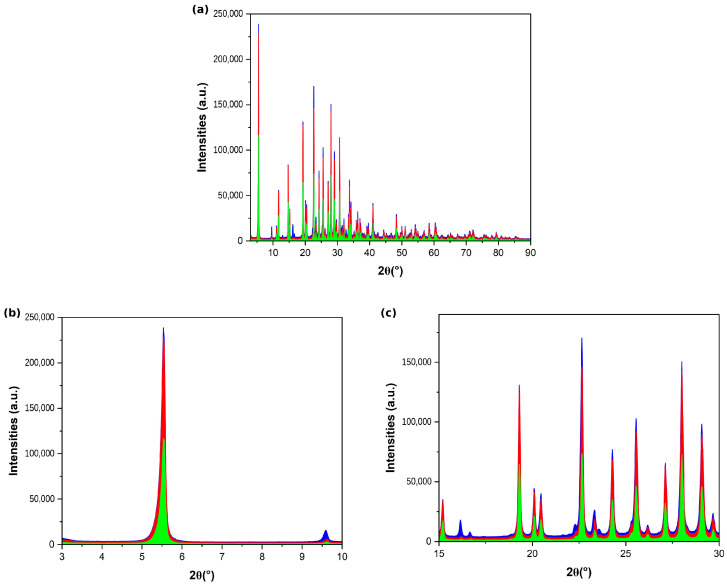
X-ray powder diffraction (XRPD) patterns of LTL zeolite for the pristine sample (green), after thallium adsorption at low concentration (red), and after thallium adsorption at high concentration (blue): (**a**) full experimental patterns; (**b**) detailed 2θ region showing 3–10°; (**c**) detailed 2θ region showing 15–30°.

The XRPD patterns of pristine K-LTL and its Tl-exchanged analogues provide clear evidence for structural modifications associated with Tl^+^ uptake within the zeolitic channels. In zeolites, the low-2θ region is particularly sensitive to extra-framework species: variations in reflection intensities within this region suggest changes in the nature and/or distribution of extra-framework cations. At the same time, shifts in peak positions indicate possible modifications of the unit-cell parameters, whereas changes in the intensities of reflections at intermediate 2θ values suggest subtle framework distortions. Thus, the diffraction data were interpreted by considering three coupled structural levels: first, Tl^+^ uptake and K^+^/Tl^+^ exchange; second, framework response through unit-cell expansion and T–O–T angular changes; and third, reorganisation of the channel water network (Figure 2).

The structural evolution of the LTL framework upon Tl^+^ exchange was initially monitored through the variation in the unit cell parameters reported in Table S1. The parent K-LTL and the low-exchange sample (K-LTL Tl low) exhibit nearly identical unit cell volumes (2201.8 Å^3^ and 2203.0 Å^3^, respectively), indicating that the treatment with the 500 ppm Tl^+^ solution induces only a very limited macroscopic strain on the aluminosilicate framework. Conversely, a more pronounced anisotropic expansion is observed for the high-exchange sample (K-LTL Tl high), where the cell volume increases by more than 22 Å^3^, reaching 2223.8 Å^3^. This expansion is associated with an increase in both the *a* parameter, 18.4428(5) Å, and the *c* parameter, 7.5494(3) Å, although the structural response is mainly expressed in the *a*/*b* plane. The increase in unit-cell volume is consistent with the incorporation of Tl^+^ into the channel system and with the larger ionic radius of Tl^+^, 1.50 Å for CN = 6, compared with K^+^, 1.38 Å for CN = 6. The refined structural model is also consistent with the elemental uptake quantified by ICP-MS, supporting the interpretation that the observed expansion is related to K^+^/Tl^+^ exchange rather than to an isolated framework effect.

The refinement quality was assessed through the agreement factors and residual profiles reported in Table S1. Despite the intrinsic complexity of modelling a partially exchanged, hydrated, multi-site system from powder diffraction data, the inclusion of Tl^+^ in the extra-framework sites led to stable refinements and chemically reasonable structural parameters. For the high-exchange sample, the weighted profile residual, R_wp_, decreases to 5.33%, compared with 7.39% for the parent K-LTL, while R_p_ decreases to 3.71%. These values support the adequacy of the proposed model for the Tl-exchanged sample, although the site assignment is not based on R-factors alone but also on Fourier difference maps, chemically reasonable Tl–O distances, refined site populations, and consistency with ICP-MS data, in addition to the use of physically meaningful occupancy constraints, as described in Section 3.6, limited correlations among site occupancy, displacement parameters, and partially overlapping cation/water positions. The parameter budget reported in Table S1 further shows that the number of refined variables, N_var_, decreases from 65 in the parent phase to 60 in the K-LTL Tl high model, indicating that the improved agreement is not the result of an increased number of refined parameters.

Selected T–O distances show slight variations, with T1–O1 shortening from 1.652 to 1.619 Å and T1–O4 increasing marginally from 1.623 to 1.629 Å, while selected T–O–T angles widen moderately, from 128° to 135° for T1–O1–T1 and from 147° to 153° for T1–O2–T1. These variations indicate minor local framework relaxation rather than a substantial deformation of the LTL framework. In this sample, Tl^+^ is not unambiguously localised by the refinement, and the extra-framework content remains largely associated with K^+^ (~9 atoms per unit cell), distributed over the KB, KC, and KD sites. This point is important because it suggests that, at 500 ppm, the structural response cannot yet be interpreted as a crystallographically resolved K^+^/Tl^+^ exchange process, but rather as an incipient perturbation of the extra-framework environment. Subtle modifications in cation coordination are observed: KB retains six-fold coordination to O3 (~2.875–2.876 Å) and O5 (~3.372 Å), maintaining an octahedral geometry; KC coordinates O4 (~3.315 Å) and O5 (~2.945 Å) with slight elongation, indicating minor polyhedral expansion; the KD site coordinates O4 and O6, with distances of approximately 3.185 Å and 3.068 Å, respectively, and also interacts with partially occupied water molecules, w1 and w2. These changes indicate an early perturbation of the cation coordination environment and of the local hydration sphere, even though Tl^+^ is not yet clearly resolved crystallographically.

**Figure 3:**
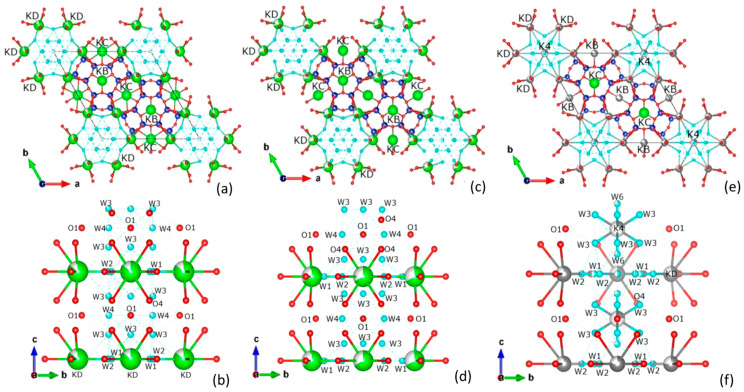
Cation and water distribution in LTL zeolite before and after thallium adsorption: (**a**) pristine LTL, viewed along the *c*-axis; (**b**) pristine LTL, side view; (**c**) low-Tl LTL, viewed along the *c*-axis; (**d**) low-Tl LTL, side view; (**e**) high-Tl LTL, viewed along the *c*-axis; (**f**) high-Tl LTL, side view. Potassium cations are shown in green, thallium cations in grey, and average water oxygen positions in cyan.

Water molecules within the channels also undergo subtle displacements: w1 shifts along x, w3 moves along x and slightly along z, and w2–w4 adjust modestly, while w5 remains essentially unchanged (Figure 3). These small rearrangements do not affect the total water content, which remains close to 28 molecules per unit cell. Therefore, after treatment with the 500 ppm Tl^+^ solution, the LTL framework retains its overall channel topology and hydration capacity, while the local cation coordination and water distribution are already slightly perturbed without significant dehydration or clearly resolved Tl^+^ occupation at specific crystallographic sites.

After treatment with the 0.5 M Tl^+^ solution, the unit-cell expansion becomes pronounced, with a = b = 18.443(5) Å, c = 7.549(3) Å, and V = 2223.8(1) Å^3^. This corresponds to a cell-volume increase of about 22 Å^3^ with respect to pristine K-LTL, from 2201.8(1) to 2223.8(1) Å^3^, i.e., a relative expansion of ~1.0%. This expansion is mainly expressed in the a/b plane, while the c parameter changes more modestly, indicating an anisotropic framework response. Consistently, the 12MR channel aperture increases from 7.50 Å in pristine K-LTL to 7.82 Å after treatment with the 0.5 M Tl^+^ solution, whereas the 8MR and 6MR windows show only minor CFA variations. This variation is consistent with the replacement of K^+^ (ionic radius = 1.38 Å for CN = 6) by the larger Tl^+^ cation (ionic radius = 1.50 Å for CN = 6), within the channel system.

**Figure 4:**
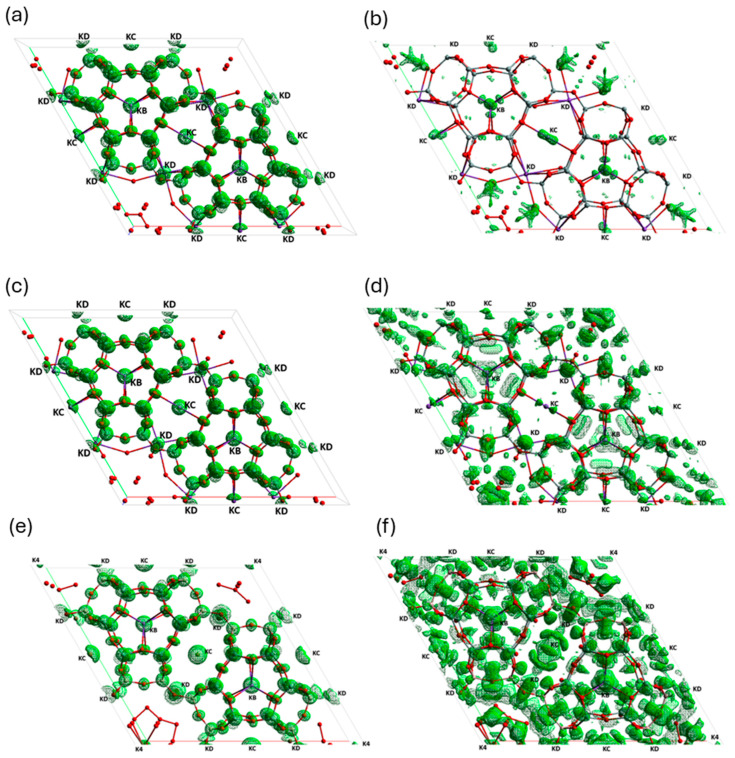
Fourier difference electron-density maps, *F_obs_* − *F_calc_*, of K-LTL, K-LTL Tl low, and K-LTL Tl high samples: (**a**) *F_obs_* K-LTL, (**b**) *F_obs_* − *F_calc_* K-LTL, (**c**) *F_obs_* K-LTL Tl low, (**d**) *F_obs_* − *F_calc_* K-LTL Tl low, (**e**) *F_obs_* K-LTL Tl high, and (**f**) *F_obs_* − *F_calc_* K-LTL Tl high.

Refinement results indicate approximately 6.95 Tl and 2 K atoms per unit cell, in the sample treated with the 0.5 M Tl^+^ solution. Considering that pristine K-LTL contains approximately nine exchangeable extra-framework K^+^ ions per unit cell, this corresponds to replacement of about three quarters of the original K^+^ population by Tl^+^. Thus, the extent of K^+^/Tl^+^ exchange is estimated directly from the refined extra-framework populations, rather than being inferred only from the adsorption experiment. This result provides the crystallographic basis for relating Tl^+^ uptake from solution to K^+^/Tl^+^ exchange within the extra-framework population. Cation distributions reveal that KB remains primarily occupied by K^+^, whereas Tl^+^ preferentially occupies KC and KD, establishing interactions with framework oxygens O4 and O6 at distances of approximately 3.28 and 3.14 Å, respectively, and contributing to the population of the newly identified K4 site (Figure 3 and Figure 4). The refined cation–oxygen and cation–water distances further support this redistribution. In K-LTL Tl high, the KB–O3 distance increases to 3.01 Å, compared with 2.88 Å in pristine K-LTL and K-LTL Tl low. Similarly, the KD–O4 and KD–O6 distances increase from 3.15 and 2.99 Å in pristine K-LTL to 3.28 and 3.14 Å in K-LTL Tl high, respectively. These variations indicate that the extra-framework cation coordination environment is modified after Tl^+^ exchange. In parallel, the cation–water environment also changes: KD–W2 increases from 2.85 Å in pristine K-LTL to 3.12 Å in K-LTL Tl high, KD–W1 is observed at 3.10 Å, and the newly populated K4 site is associated with K4–W3 and K4–W6 contacts at 2.53 and 2.10 Å, respectively. These changes support the interpretation that Tl^+^ exchange is accompanied by reorganisation of both cation–framework and cation–water coordination environments. Therefore, Tl^+^ exchange does not simply reproduce the initial K^+^ distribution but induces a redistribution of the extra-framework cations toward the more accessible channel sites. This cation redistribution is accompanied by framework deformation. T–O distances near cation sites exhibit both contraction and elongation: T1–O1 and T1–O2 contract, whereas T2–O5 elongates. At the same time, selected T–O–T angles increase markedly, with T1–O2–T1 reaching 168°and T2–O3–T2 reaching 150°, reflecting channel-specific tetrahedral opening. The increase in selected T–O–T angles by up to ~20° with respect to pristine K-LTL quantitatively indicates significant local framework distortion. This response is mainly expressed through angular opening of selected oxygen bridges rather than through a uniform elongation of all T–O bonds, confirming that Tl^+^ exchange induces a localised relaxation of the LTL framework while preserving its topology.

The water content decreases from ≈28 to ≈21 H_2_O per unit cell, corresponding to a decrease of about 25%. This reduction is structurally coupled with Tl^+^ incorporation and is not simply attributable to the loss of physisorbed water. The water molecules w1 and w2 shift positions and decrease in occupancy, from 0.90 to 0.50 for w1 and from 1.00 to 0.89 for w2. These changes are accompanied by longer cation–water distances, approximately 3.1–3.2 Å, indicating weakened hydration of the residual K^+^ population. The intersection site w4 is emptied, while new hydration environments appear: w3 remains fully occupied but is displaced by *Δx* ≈ −0.11, *Δy* ≈ +0.12, and *Δz* ≈ +0.14, and a new water site, w6, is detected with partial occupancy, approximately 0.70, in association with the newly populated K4 site.

Taken together, the decrease in total water content, the reduced occupancies of w1 and w2, the emptying of w4, the displacement of w3, and the appearance of w6 provide crystallographic evidence for reorganisation of the channel water network. The substitution of approximately 6.95 Tl^+^ per unit cell is therefore structurally coupled with partial dehydration and substantial redistribution of the extra-framework water arrangement, indicating a competitive interaction between Tl^+^ and channel water for coordination space. The formation of Tl–O contacts at KC and KD indicates a shift from water-mediated K^+^ stabilisation to more direct Tl^+^–framework coordination. The combined occurrence of (i) anisotropic unit-cell expansion, approximately 1.0%, (ii) replacement of about three quarters of the original K^+^ population by Tl^+^, (iii) approximately 25% reduction in channel water, and (iv) significant T–O–T angular opening provides quantitative support for describing Tl^+^ as a structure-breaking cation with respect to the pre-existing K^+^-associated hydration environment [40,41,42]. In this context, “structure-breaking” refers to disruption and redistribution of the K^+^-associated hydration environment, as reflected by changes in refined water-site occupancies and positions, not to degradation of the aluminosilicate framework. This behaviour is linked to the high polarisability of Tl^+^, which favours direct framework coordination and perturbs the long-range organisation of hydration shells typically associated with K^+^.

Pore-aperture analysis reveals a differential structural response of the LTL framework to increasing Tl^+^ uptake. The 12MR channels undergo a significant expansion, from 7.50 Å in pristine K-LTL to 7.68 Å after treatment with the 500 ppm Tl^+^ solution, reaching 7.82 Å after treatment with the 0.5 M Tl^+^ solution. This widening is reflected in the crystallographic free area (CFA, *sensu* Baerlocher, calculated as *π* × *r^2^*, where *r* is the mean channel radius), which increases by approximately 9%, from 44.11 to 48.04 Å^2^ [27]. Together with the unit-cell expansion mainly observed in the *a*/*b* plane, this increase in CFA provides quantitative evidence for anisotropic expansion of the 12MR channel system. This quantitative increase demonstrates that the main 12MR channels represent the most responsive region of the LTL pore system and the principal structural pathway for Tl^+^ accommodation.

By contrast, the smaller 8MR and 6MR windows exhibit only minor changes in CFA, from 10.79 to 10.62 Å^2^ for the 8MR windows and from 4.55 to 4.97 Å^2^ for the 6MR windows, indicating that these apertures remain comparatively rigid. The channel ellipticity, ε, defined as the ratio of the longest to shortest O–O distances, remains nearly constant, ε ≈ 1.05–1.06. Therefore, the 12MR channels expand without a major change in aperture shape. This selective enlargement of the 12MR channels, compared with the limited response of the 8MR and 6MR windows, supports the interpretation that Tl^+^ accommodation occurs mainly within the principal LTL channel system, while the smaller windows contribute less to the structural response.

This structural interpretation is consistent with the high Tl^+^ removal efficiency observed experimentally at low Tl^+^ concentration, approximately 98%. Although the most pronounced crystallographic changes are observed after treatment with the 0.5 M Tl^+^ solution, the adsorption data show that K-LTL removes Tl^+^ efficiently under dilute conditions. The structural results suggest that Tl^+^ uptake is favoured by the capacity of the LTL framework to combine cation exchange, partial dehydration, and channel expansion while preserving crystallinity.

To further investigate the structural modifications induced by Tl^+^ incorporation, Fourier difference maps, *F_obs_* − *F_calc_*, derived from Rietveld refinement were analysed to evaluate the electron-density distribution in pristine K-LTL, K-LTL Tl low, and K-LTL Tl high. As shown in Figure 4, clear differences emerge among the three samples. In the K-LTL Tl high sample, new intense electron-density maxima are observed at extra-framework cationic positions, consistent with the high scattering power of Tl^+^, Z = 81, while electron density decreases at selected hydration sites. These features support the assignment of Tl^+^ to redistributed extra-framework sites and show that cation exchange is accompanied by modification of the channel water distribution.

In the K-LTL Tl high sample, the redistribution of water molecules around the cationic sites is accompanied by the appearance of a new cationic site, K4, associated with a region previously occupied by the w5 water site. The depletion of electron density at original hydration sites and the emergence of new intense maxima at Tl^+^ positions indicate that the extra-framework region is reorganised after Tl^+^ exchange. By contrast, the K-LTL Tl low sample exhibits only minor deviations from pristine K-LTL, although a partial rearrangement of the water network is already evident. This distinction is important: after treatment with the 500 ppm Tl^+^ solution, the structural response is limited to subtle framework and water-site perturbations, whereas after treatment with the 0.5 M Tl^+^ solution, Tl^+^ is crystallographically resolved and the extra-framework population is substantially reorganised.

A significant feature is therefore the disruption of the pre-existing K^+^-centred coordination and hydration environment. Upon entering the KC and KD sites in place of K^+^, Tl^+^ modifies the local extra-framework arrangement, establishes direct interactions with framework oxygen atoms, and promotes redistribution of nearby water molecules. This behaviour can be described as a structure-breaking effect with respect to the K^+^-associated hydration environment. In this context, “structure-breaking” does not refer to degradation of the aluminosilicate framework, which remains crystalline, but to the disruption and redistribution of the water-site arrangement associated with K^+^ hydration within the LTL channels. This interpretation is consistent with the crystallographic evidence for partial dehydration, channel expansion, cation-site redistribution, and modification of the refined water-site distribution.

**Figure 5:**
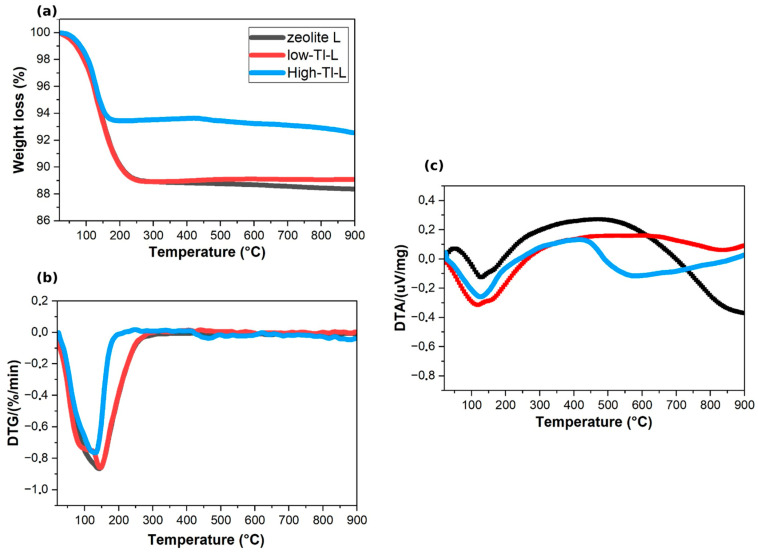
Thermal analyses of the three samples: (**a**) Thermogravimetric analysis (TGA), (**b**) derivative thermogravimetry (DTG) and, (**c**) differential thermal analysis (DTA). The black line corresponds to the K-LTL sample, the red line to K-LTL Tl low, and the blue line to K-LTL Tl high.

Thermal analyses (TG, DTG, and DTA) further support the structural observations. The as-synthesised L exhibits the largest weight loss (~12–13%) with sharp DTG and DTA peaks, consistent with fully hydrated channels. Low-Tl-L shows slightly lower weight loss (~10–11%) and pronounced endotherms at 150–200 °C, indicative of water removal from K^+^ sites. High-Tl-L displays even smaller weight loss (~8–9%), broader DTG peaks, and weaker DTA signals shifted to higher temperatures, indicating a broader distribution of dehydration environments rather than a single dominant water-release process (Figure 5). Specifically, the DTG profile of High-Tl-L shows a widening of the main dehydration event, which extends over a larger temperature range compared to the sharper and more defined peak observed for the pristine and low-Tl samples. This broadening indicates that water molecules are released from multiple energetically distinct environments rather than from a single homogeneous hydration state. In addition, the maximum of the DTG/DTA dehydration signal is shifted toward higher temperatures, consistent with an increased fraction of water molecules more strongly bound to extra-framework cations, particularly in Tl-rich coordination environments compared to K^+^-dominated sites. The progressive decrease in total mass loss, together with the broadening of the DTG dehydration interval and the shift of thermal features toward higher temperature in High-Tl-L, further supports the interpretation that water is no longer released from a single, relatively uniform hydration environment. Instead, dehydration occurs over a wider temperature range, consistent with fewer water molecules being distributed over distinct crystallographic hydration sites after Tl^+^ exchange. These thermal changes are consistent with the crystallographic evidence for partial dehydration and redistribution of water molecules into fewer, structurally distinct environments after Tl^+^ exchange. In particular, the thermal data support the decrease in refined water content from approximately 28 to 21 H_2_O molecules per unit cell, the reduced occupancies of w1 and w2, the emptying of w4, the displacement of w3, and the appearance of the new w6 water site. Therefore, the thermal behaviour provides independent evidence that the extra-framework water network is reorganised rather than simply depleted.

Overall, progressive K^+^ → Tl^+^ exchange drives a coupled structural response characterised by anisotropic channel expansion, selective cation redistribution, and reorganisation of extra-framework water. Each of these features is supported by specific crystallographic evidence. Anisotropic channel expansion is demonstrated by the unit-cell variation and by the increase in 12MR CFA from 44.11 to 48.04 Å^2^. Selective cation redistribution is shown by the preferential location of Tl^+^ at KC and KD, while KB remains mainly K-rich and K4 appears as an additional cationic site. Water-network reorganisation is supported by the decrease in water content, the reduced occupancies of w1 and w2, the emptying of w4, the displacement of w3, and the appearance of w6.

After treatment with the 500 ppm Tl^+^ solution, only subtle modifications of framework geometry and water positions are observed, while Tl^+^ is not unambiguously localised by refinement. In contrast, after treatment with the 0.5 M Tl^+^ solution, Tl^+^ exchange significantly alters both the occupancy and spatial distribution of extra-framework water molecules, producing a modified hydration-site distribution within the channels. These results support the interpretation that Tl^+^ acts as a structure-breaking cation with respect to the K^+^-associated extra-framework hydration environment, because it replaces water-mediated K^+^ stabilisation with more direct Tl^+^–framework coordination and induces redistribution of the channel water sites. This interpretation does not imply loss of crystallinity or degradation of the LTL framework; rather, it describes the reorganisation of cations, framework apertures, and hydration sites within a structurally preserved zeolitic host.

### 2.3. Batch Adsorption Kinetics and Isotherms

To investigate the adsorption kinetics and equilibrium behaviour of the material toward Tl, batch adsorption experiments were performed by measuring the residual thallium concentration in the aqueous solution after contact with the solid phase.

The obtained results (Table S3) indicate that thallium removal is essentially independent of contact time within the investigated range. The removal efficiency remained nearly constant at approximately 99.5% from 5 to 1440 min. Similarly, the equilibrium adsorption capacity (q_e_ ~ 2.40 mg g^−1^) and the equilibrium concentration (C_e_ ~ 1.0 ppm) showed negligible variation over time. These findings demonstrate that adsorption reaches equilibrium very rapidly, within the first few minutes of contact, thus highlighting the high efficiency and fast uptake kinetics of Tl by LTL zeolite.

Regarding the adsorption isotherms, the solid-to-liquid ratio plays a key role in controlling Tl removal from solution. The experimental q_e_–C_e_ data were fitted using four adsorption models: Langmuir isotherm, Freundlich isotherm, Temkin isotherm and Harkins–Jura isotherm [43].

The fitting parameters and statistical coefficients are summarised in Table 3.

**Figure 6:**
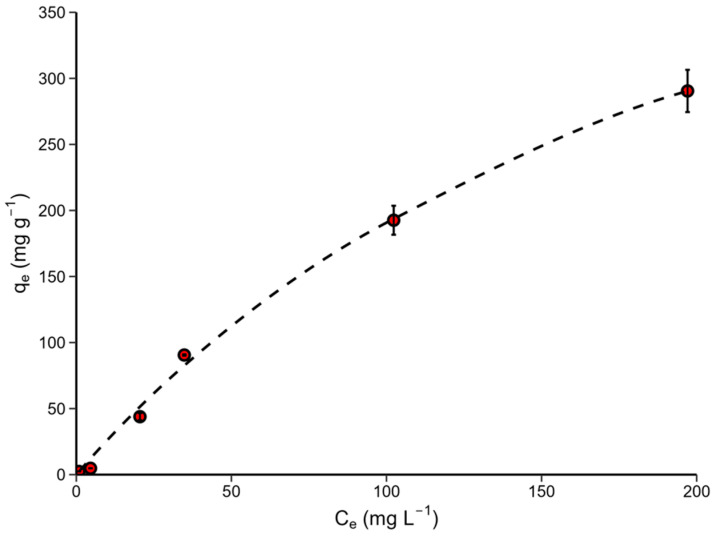
Experimental adsorption isotherm of Tl^+^ onto K-L zeolite (q_e_ vs. C_e_). Symbols represent experimental data (mean ± standard deviation) obtained from three replicates for each sample, while the dashed line corresponds to the nonlinear Langmuir model fit (R^2^ = 0.99).

Among the tested models, the Langmuir isotherm [42] provided the best fit to the experimental data (Figure 6), with a *p*-value < 0.01, R^2^ = 0.99, and the lowest AIC value (43.3). The calculated maximum adsorption capacity (q_max_) was 631 mg g^−1^, with a Langmuir constant (KL) of 0.004 L g^−1^. These values are specific to the experimental conditions used for K-LTL, namely near-neutral pH (~7), a solid-to-liquid ratio of 0.2 g mL^−1^, and an initial Tl^+^ concentration of 500 ppm. Comparable Langmuir-based adsorption studies for Tl^+^ removal reported in the literature also support the applicability of the Langmuir model for Tl adsorption systems, where monolayer adsorption on a finite number of active sites is typically observed [44,45,46]. These values are model-derived parameters and depend on the specific experimental conditions; therefore, the q_max_ value should not be interpreted as an intrinsic adsorption capacity. This variability is also reflected in Table 1.

These results indicate that Tl adsorption onto LTL zeolite is well described by a monolayer adsorption process occurring on a relatively homogeneous surface with a finite number of identical active sites. The calculated q_e_ value reflects the strong adsorption capacity of the material toward thallium under the investigated experimental conditions, supporting its potential applicability for Tl removal from aqueous solutions. However, comparison of adsorption capacities across different studies and direct comparison of q_max_ values among different adsorbents should be undertaken with caution, as these values are strongly dependent on experimental conditions such as initial concentration, pH, solid-to-liquid ratio, and adsorption protocol.

## 3. Materials and Methods

### 3.1. Materials

Thallium solutions were prepared using thallium(I) nitrate (TlNO_3_, 99.999% purity, Sigma-Aldrich, (Merck Life Science GmbH, Darmstadt, Germany), Catalogue No. 204609) and grade 1 water (arium^®^mini plus UV, Sartorius, Varedo (MB), Italy). The pH of solutions was adjusted using hydrochloric acid (HCl, 4%) and potassium hydroxide (KOH, 3%) (purity details provided in the specifications). Nitric acid (HNO_3_, 67–70% suprapure for trace elements, Carlo Erba Reagents, Milan, Italy) was used for acidification of the water employed in ICP-MS analyses.

The material selected for the adsorption experiments was synthetic, commercial large-pore zeolite L, purchased from the Tosoh Corporation (Tokyo, Japan; HSZ-500KOA code), in its potassium form. This sample has a SiO_2_/Al_2_O_3_ ratio of 6.1, a Na_2_O content of 0.25 wt%, and a surface area of 290 m^2^/g. The same sample was previously used in the studie by Precisvalle et al. (2023) [34], employing synchrotron and neutron diffraction techniques.

### 3.2. Batch Adsorption and Ion-Exchange Experiments

For the adsorption experiments, the following procedures were followed.

A 500 ppm thallium solution was prepared by dissolving approximately 17 mg of TlNO_3_ in 25 mL of grade 1 water, which was stirred for about 3 h. The pH was adjusted to 7.43. For these samples, the removal efficiency was also calculated as a function of pH, with additional solutions at the same concentration prepared at pH 3.04, 5.33, and 9.48. In a 2 mL Eppendorf tube, an aliquot of the thallium solution and 0.2 g of K-L zeolite were combined. The mixture was mechanically stirred on an orbital shaker at approximately 150 rpm for 24 h at room temperature. After centrifugation at 4000 rpm for 10 min, the supernatant was collected for ICP-MS analysis, while the solid fraction was dried in a fume hood for three days before being used for XRPD and thermal analyses.

For the 0.5 M thallium solution, a more concentrated solution was prepared by dissolving 5.3 g of TlNO_3_ in 40 mL of grade 1 water, stirred for 5 h, and adjusted to pH 7.16. To 10 mL of this solution, 0.5 g of K-L zeolite was added, and the mixture was mechanically stirred on an orbital shaker at approximately 150 rpm at room temperature for 24 h. After centrifugation, the supernatant was stored, and the solid phase was washed twice with grade 1 water followed by centrifugation. The resulting solid was then dried and used for XRPD, thermal, and ICP-MS analyses.

### 3.3. Adsorption Isotherms

Adsorption isotherms were determined at 25 °C using six different solid-to-liquid (S/L) ratios ranging over two orders of magnitude (from 1 to 200). Accordingly, variable amounts of K-L zeolite (from 0.001 g to 0.2 g) were added to 1 mL of a 485 mg L^−1^ thallium solution in 2 mL Eppendorf tubes. All experiments were conducted at pH 7, which was adjusted and maintained prior to the adsorption tests. The suspensions were shaken on an orbital shaker at approximately 150 rpm for 24 h to ensure equilibrium conditions. After centrifugation at 4000 rpm for 10 min, the supernatant was carefully collected and analysed by ICP-MS to determine the residual thallium concentration. The amount of thallium adsorbed at equilibrium (q_e_, mg g^−1^) [47] was calculated using a mass-balance approach according to:

$$q_{e}=\frac{(C_{0}-C_{e})V}{m}$$

where $C_{0}$ and $C_{e}$ represent the initial and equilibrium thallium concentrations (mg L^−1^), respectively, V is the solution volume (L), and m is the mass of K-L zeolite (g). In addition, the removal efficiency (R_E_, %) [48] was calculated to evaluate the percentage of thallium removed from solution, according to:

$$R_{E}(\%)=\frac{C_{0}-C_{e}}{C_{0}}\times 100$$

This parameter provides a direct measure of the effectiveness of the adsorption process under the investigated experimental conditions.

To describe the adsorption behaviour and elucidate the interaction mechanisms between thallium and the zeolitic surface, the experimental data were fitted using four different isotherm models: Langmuir, Freundlich, Temkin, and Harkins–Jura [43].

The Langmuir model assumes monolayer adsorption onto a homogeneous surface with a finite number of identical active sites and is expressed in its nonlinear form as [49]:

$$q_{e}=\frac{q_{max}K_{L}C_{e}}{1+K_{L}C_{e}}$$

where $q_{max}$ represents the maximum adsorption capacity and $K_{L}$ is the Langmuir affinity constant.

The Freundlich model, which describes multilayer adsorption on heterogeneous surfaces [50], is given by:

$$q_{e}=K_{F}C_{e}^{1/n}$$

where $K_{F}$ is related to adsorption capacity and n is an empirical parameter describing surface heterogeneity.

The Temkin model accounts for adsorbate–adsorbent interactions and assumes that the heat of adsorption decreases linearly with increasing surface coverage. Its nonlinear form is [49]:

$$q_{e}=\frac{RT}{b_{T}}\ln(K_{T}C_{e})$$

where R is the universal gas constant, *T* is the absolute temperature, $K_{T}$ is the Temkin equilibrium constant, and $b_{T}$ is related to the heat of adsorption.

Finally, the Harkins–Jura model, which describes multilayer adsorption on heterogeneous pore surfaces, was applied in its nonlinear form as [51]:

$$q_{e}={\left(\frac{A}{b-log(C_{e})}\right)}^{1/2}$$

where A and b are empirical constants related to surface heterogeneity and pore distribution.

All experimental data were processed using RStudio (version 2026.01.0+392) [52], and nonlinear regression analyses were performed using the PUPAIM package (version 0.3.1) [53] to estimate model parameters. The goodness of fit of each model was evaluated through the coefficient of determination (R^2^), the Akaike Information Criterion (AIC), and the statistical significance of the model parameters (*p*-values). The best-fitting isotherm model was selected based on the highest R^2^, the lowest AIC value, and statistically significant parameter estimates.

### 3.4. Adsorption Kinetics

Adsorption kinetics were investigated to evaluate the rate of thallium uptake by K-L zeolite and to identify the controlling adsorption mechanisms. Batch experiments were carried out at 25 °C using a fixed initial thallium concentration (485 ppm) under the same experimental conditions described for the isotherm studies (pH 7, orbital shaking at approximately 150 rpm).

At predetermined contact times (5, 10, 30, 60, 120, and 1440 min), triplicate samples were centrifuged at 4000 rpm for 10 min, and the residual thallium concentration in the supernatant (*C_t_*) was determined by ICP-MS.

The amount of thallium adsorbed at time t (*q_t_*, mg g^−1^) was calculated using the same mass-balance equation adopted for equilibrium conditions:

$$q_{t}=\frac{(C_{0}-C_{t})V}{m}$$

where $C_{0}$ is the initial concentration, $C_{t}$ is the concentration at time t, V is the solution volume, and m is the mass of K-L zeolite. The removal efficiency at time t (*R_E_*, %) was calculated analogously to the equilibrium removal efficiency, according to:

$$R_{E}(\%)=\frac{C_{0}-C_{t}}{C_{0}}\times 100$$

### 3.5. X-Ray Powder Diffraction (XRPD)

Data collection was performed using a Bruker D8 Advance Da Vinci diffractometer (Bruker, Billerica, MA, USA) operating in Bragg–Brentano geometry. The instrument, located in the Department of Physics and Earth Sciences of the University of Ferrara (Italy), is equipped with an X-ray tube with a copper anode and a Lynx-Eye XE silicon strip detector with an angular coverage range of 2.946° 2θ, calibrated to discriminate Cu Kα1,2 radiation. The sample was laterally loaded into a 2 mm deep cavity within a polymethylmethacrylate (PMMA) sample holder. The scan was conducted at room temperature (RT) in continuous mode from 3 to 90° 2θ, with a step size of 0.02° 2θ and a counting time of 3 s per step. In addition, a blade perpendicular to the sample holder was positioned at a sub-millimetre distance to reduce air scattering at low angles.

### 3.6. Rietveld Refinement and Structural Modelling

Rietveld refinements were performed using the GSAS software package (Los Alamos National Laboratory, USA, last download June 2026) and the EXPGUI graphical interface [54,55] in the hexagonal space group P6/mmm starting from the structural model reported by Refs. [56,57]. Peak profiles were modelled using the Pseudo-Voigt function, with a 0.0025% cut-off intensity. The refined profile parameters included the Gaussian broadening terms (GW and GV), the Lorentzian components (LY and LX), and an axial divergence asymmetry correction. A 10-term Chebyshev polynomial function was employed to fit the instrumental background. The scale factor and 2θ zero shift were subsequently refined to ensure precise agreement between the observed and calculated patterns.

To resolve the extra-framework configurations, difference Fourier electron-density maps were calculated iteratively. Initial soft constraints on the tetrahedral coordination framework (Si-O = 1.64 Å, σ = 0.04 and Al-O 1.72 Å, σ = 0.04) were relaxed during the advanced stages of refinement. To maintain physically meaningful extra-framework populations during the refinement of partially occupied and closely spaced K^+^, Tl^+^, and H_2_O sites, the total site occupancy factors, SOFs, were constrained according to site multiplicity, site capacity, and chemically reasonable local configurations. Charge-balance consistency was evaluated by comparing the refined extra-framework populations with the framework composition and the ICP-MS-derived elemental ratios. In addition, isotropic atomic displacement parameters, U_iso_, of closely spaced or partially overlapping extra-framework cation and water sites were linked when necessary to avoid non-physical divergence of correlated occupancy and displacement parameters.

The assignment of extra-framework Tl^+^ sites was based on four complementary criteria: residual electron-density maxima in the difference Fourier maps, the massive X-ray scattering contrast of thallium (Z = 80) compared to potassium (Z = 19) and oxygen (Z = 8), chemically reasonable Tl–O distances consistent with the larger ionic radius of Tl^+^ (1.50 Å) over K^+^ (1.38 Å), and consistency between refined site populations and ICP-MS chemical data. In the sample treated with the 0.5 M Tl^+^ solution, the strongest residual electron-density maxima occur at accessible channel sites, mainly KC and KD, whereas no unambiguous Tl^+^ site could be refined for the sample treated with the 500 ppm Tl^+^ solution. The refined population of approximately 6.95 Tl atoms and 2 residual K atoms per unit cell corresponds to the replacement of about three quarters of the original exchangeable K^+^ population, in agreement with the Tl/K ratio obtained from ICP-MS within the uncertainty expected for powder diffraction refinement of hydrated extra-framework species.

The final refinement quality was evaluated using standard profile residuals, R_wp_ and R_p_, goodness of fit, χ^2^, and inspection of the residual profiles. Detailed data collection parameters, final R-factors, refinement statistics, and occupancy constraints for all samples are summarised in Tables S1 and S2 in the Supplementary Materials; the corresponding crystallographic information files, CIFs, are also provided. The agreement factors, chemically reasonable bond distances, residual electron-density maps, and consistency between Rietveld-derived populations and ICP-MS data support the robustness of the proposed structural models.

### 3.7. Thermal Analysis

Thermogravimetric (TGA) and differential thermal (DTA) analyses were conducted using a Netzsch STA 409 PC LUXX^®^ simultaneous TG/DTA instrument (Netzsch Gerätebau, Selb, Germany). Measurements were performed in a controlled atmosphere of synthetic air, from room temperature up to 1000 °C, at a constant heating rate of 10 °C/min. Approximately 30 mg of sample was used for each measurement. An alumina (Al_2_O_3_) reference standard was employed to ensure baseline stability and accurate DTA signal calibration.

### 3.8. Inductively Coupled Plasma Mass Spectrometry (ICP-MS) Analysis

The thallium and potassium concentration in the liquid phase, before and after treatment, was determined by inductively coupled plasma mass spectrometry (ICP-MS) using an iCAP-TQ ICP-MS spectrometer (Thermo Scientific, Waltham, MA, USA). The samples were diluted 1:1000 (*v*:*v*) with grade 1 water acidified with 2% (*v*:*v*) HNO_3_ to fall within the instrument’s calibration range.

For solid samples, thallium and potassium were determined by ICP-MS after acid digestion. Specifically, 0.2 g of powdered sample was placed into a PTFE vessel and digested in a Multiwave 5000 microwave system equipped with a 20SVT50 rotor (Anton Paar GmbH, Graz, Austria) using 6 mL of suprapure hydrofluoric acid and 3 mL of suprapure nitric acid (both Suprapure, Carlo Erba Reagents, Milan, Italy). The digestion program consisted of a 10 min ramp up to 180 °C, followed by a 25 min hold at this temperature, and subsequent cooling, with a total duration of about 1 h. After digestion, the solutions were transferred into PTFE beakers and evaporated on a hot plate at ~180 °C until near dryness. The residues were then re-dissolved in 2 mL of nitric acid and diluted to a final volume of 100 mL with grade 1 water.

From the experimental data, the removal efficiency (R_e_%) was calculated for each sample as the ratio between the amount of thallium adsorbed by the zeolite and the initial thallium concentration, multiplied by 100. The adsorption capacity (q_e_, mg g^−1^) was also determined using the following equation: (initial thallium concentration − final thallium concentration in solution) × 10^−4^/mass of the material used.

### 3.9. Statistical Analysis

To evaluate whether pH had a significant effect on q_e_ and R_e_%, one-way ANOVA followed by a Tukey HSD test (at *p* = 0.05) was carried out. Data normality and homoscedasticity were first tested with Shapiro–Wilk and Levene’s tests to verify the assumptions of ANOVA. When these assumptions were not met, the non-parametric Kruskal–Wallis test was applied instead. R (4.5.1) and R studio (2026.01.1 build 403 [52]) software were employed for statistical analysis using the Agricolae package [58].

## 4. Conclusions

This study investigates the potential of potassium-form L zeolite, K-LTL, for the removal of thallium, Tl^+^, from aqueous solutions, with particular attention to the structural and extra-framework changes associated with Tl^+^ uptake. The combined adsorption, ICP-MS, XRPD/Rietveld, Fourier difference map, and thermal data indicate that Tl^+^ removal is associated with K^+^/Tl^+^ exchange within the LTL channel system. At low Tl^+^ concentration, the structural response is limited to minor adjustments around extra-framework cation sites and subtle changes in the channel water site distribution. In contrast, treatment with a 0.5 M Tl^+^ solution produces a more pronounced crystallographic response, including anisotropic expansion of 12MR channels, selective redistribution of extra-framework cations, partial dehydration, and modification of refined water site distribution. In this specific sense, Tl^+^ exerts a structure-breaking effect on the K^+^-associated hydration environment, by favouring more direct Tl^+^–framework coordination and redistributing channel water sites, without loss of crystallinity of the LTL framework. These findings show that Tl^+^ uptake by K-LTL cannot be described only as a solution-based adsorption process, but involves a coupled structural response of the zeolite host.

Adsorption experiments confirm the high affinity of K-LTL for Tl^+^ under the investigated conditions. Batch tests performed at near-neutral pH showed rapid Tl^+^ uptake, with a high removal efficiency already reached within the first few minutes and maintained over the investigated contact times. Because the time-dependent data show little variation in C_e_ and q_e_ after the first measured interval, the results indicate rapid attainment of apparent equilibrium under the tested conditions; however, no detailed kinetic model was applied. Equilibrium isotherm analysis was best described by the Langmuir model, yielding a calculated maximum adsorption capacity of *q_max_* = 631 mg g^−1^. This value should be interpreted as a model-derived maximum capacity under the experimental conditions used in this study, rather than as a universal performance parameter. Nevertheless, together with the high removal efficiency and the crystallographic evidence for Tl^+^ incorporation into extra-framework sites, the adsorption results support the relevance of K-LTL as a crystalline ion-exchange material for Tl-contaminated waters.

Future work will focus on regeneration and reuse tests to evaluate the stability of the Tl^+^-exchanged material, the reversibility of the ion-exchange process, and the preservation of the zeolite framework after multiple adsorption–desorption cycles. Assessing regeneration efficiency, possible Tl^+^ release, and structural integrity after repeated use will be essential to determine the long-term applicability of K-LTL for practical water treatment applications.

## Figures and Tables

**Table 1:** Comparison of representative thallium adsorbents reported in the literature, including the K-LTL zeolite studied in this work, showing adsorption capacity (q_max_), removal mechanism, and regeneration/stability characteristics.

Adsorbent Category	Examples (References)	q_max_ (mg g^−1^)	Removal Mechanism	Regeneration and Stability
Zeolite K-LTL (this work)	K-LTL	631	1D ion exchange (Tl^+^/K^+^ isomorph)	Preserved crystalline framework after Tl^+^ uptake; regeneration and long-term cycling require dedicated assessment
Hybrid systems	18-crown-6 @ K-4A [18]	1199.0	Ion exchange + chelation (crown ether)	Complex; risk of leaching of expensive/toxic organic components
Manganese oxides	Amorphous or “flower-like” MnO_2_ [19,20,21]	320.0	Surface adsorption/redox	Limited; rapid saturation of external layer; risk of aggregation or acid dissolution
Industrial residues (WTR)	Fe-WTR/Al-WTR [22]	0.69–3.75	Surface complexation	Poor; too low capacity for large-scale use; labile bonds sensitive to pH
Nanomaterials	Carbon nanotubes [23], nano-Al_2_O_3_ [24]	Variable	Physical/electrostatic adsorption	Difficult; high recovery cost; risk of environmental nano-pollution
Biomass	Sawdust [25], beetroot [26]	<20.0	Non-specific interactions	Absent; single-use materials; biological degradation and release of COD/tannins

**Table 2:** Experimental conditions and results for untreated and Tl-exchanged LTL zeolites. The table reports solution parameters (volume, pH, and initial Tl concentration), post-treatment Tl concentrations in both liquid and solid phases, removal efficiency, and Tl content estimated by Rietveld refinement.

	K-LTL	K-LTL Tl Low	K-LTL Tl High
Solution volume (mL)	-	1	10
Material mass (g)	-	0.2	0.5
pH	-	7.43	7.16
Initial liquid Tl concentration (ppm)	-	508	102,190
Post-treatment liquid Tl concentration (ppm)	-	10	Not detected
Post-treatment solid Tl concentration (ppm)	<0.01	2155	346,889
Removal efficiency (%)	-	98	16.9 (estimated)
Adsorption capacity (q_e_, mg g^−1^)	-	2.48	345.4
Rietveld refinement Tl estimated concentration (ppm)	-	3500	336,000

**Table 3:** Regression parameters and statistical coefficients derived from nonlinear fitting of the experimental data to the Freundlich isotherm, Langmuir isotherm, Temkin isotherm, and Harkins–Jura isotherm models.

Freundlich					
T [K]	R^2^	*p*-value	KF [mg⋅g^−1^⋅(L⋅g^−1^)1/n]	*n*	AIC
298.15	0.99	0.00001798	5.91	1.35	49.9
Langmuir					
T [K]	R^2^	*p*-value	q_max_ [mg⋅g^−1^]	K_L_ [L⋅g^−1^]	AIC
298.15	0.99	0.000002065	631	0.004321	43.3
Temkin					
T [K]	R^2^	*p*-value	AT [L⋅g^−1^]	bT [J⋅mol^−1^]	AIC
298.15	0.73	0.01949	0.403	4.11	69.8
Harkins–Jura					
T [K]	R^2^	*p*-value	α [g^2^⋅mg^−2^]	β	AIC
298.15	0.98	0.001268	396	5.95	63.4

## Data Availability

The original contributions presented in this study are included in the article/Supplementary Materials. Further inquiries can be directed to the corresponding authors.

## Supplementary Materials

The following supporting information can be downloaded at: https://www.mdpi.com/article/10.3390/molecules31122130/s1, Table S1: Structural refinement parameters of the LTL samples; Table S2: Framework and extra-framework distances of K-LTL, K-LTL Tl low (500 ppm) and K-LTL Tl high (0.5 M) samples; Table S3: Adsorption kinetics obtained using 0.200 g of zeolite LTL and 1 mL of thallium solution with an initial concentration of 485 ppm. *C_e_* = equilibrium concentration, q_e_ = adsorption capacity at equilibrium, *R_E_* = removal efficiency; Figure S1: Observed and calculated diffraction patterns and final difference curve from Rietveld refinement of K-LTL; Figure S2: Observed and calculated diffraction patterns and final difference curve from Rietveld refinement of K-LTL Tl low; Figure S3: Observed and calculated diffraction patterns and final difference curve from Rietveld refinement of K-LTL Tl high.
