# Supplementary material for: Thallium Removal from Aqueous Solutions Using L Zeolite: Structural Modifications, Cation Distribution and Water Network Reorganisation
Source: Molecules. 2026 Jun 17;31(12):2130. doi: 10.3390/molecules31122130 (PMC13305177; doi:10.3390/molecules31122130)
Supplement: Supplementary file 1 [file molecules-31-02130-s001.zip › molecules-4353405-supplementary.pdf]

Supplementary Materials of

Thallium Removal from Aqueous Solutions Using L Zeolite: Structural Modifications, Cation Distribution and Water Network Reorganisation

Luca Adami [1,*], Maura Mancinelli [1], Francesco Di Benedetto [1], Renzo Tassinari [1], Matteo Alberghini [2], Giacomo Ferretti [3] and Annalisa Martucci [1,*]

[1] Department of Physics and Earth Sciences, University of Ferrara, Via Saragat 1, 44122 Ferrara, Italy

[2] Department of Environmental and Prevention Sciences, University of Ferrara, Via Borsari 46, 44121 Ferrara, Italy

[3] Department of Chemical, Pharmaceutical and Agricultural Sciences, University of Ferrara, Via Borsari 46, 44121 Ferrara, Italy

Table S1 Structural refinement parameters of the LTL samples.

Unit cell parameters	K-LTL	K-LTL Tl low	K-LTL Tl high
a (Å)	18.3778(4)	18.3808(5)	18.4428(5)
b (Å)	18.3778(4)	18.3808(5)	18.4428(5)
c (Å)	7.5278(2)	7.5293(2)	7.5494(3)
α (°)	90	90	90
β (°)	90	90	90
γ (°)	120	120	120
Cell Volume (Å^3)	2201.8(1)	2203.0(1)	2223.8(1)
R_{wp}(%)	7.39	7.54	5.33
R_p(%)	4.58	4.61	3.71
R_{F^2}(%)	8.21	8.66	5.96
N_{obs}	4252	4252	4259
N_{var}	65	63	60
χ^2	1.7	1.8	1.4

Table S2 Framework and extraframework distances of K-LTL, K-LTL Tl low (500 ppm) and K-LTL Tl high (0.5M) samples.

Bond distances (Å)	K-LTL	K-LTL Tl low	K-LTL Tl high
T1-O1	1.652(9)	1.619(4)	1.629(5)

T1-O2	1.633(9)	1.600(4)	1.577(4)
T1-O4 [X2]	1.623(9)	1.629(3)	1.668(4)
**<T1-O>**	1.633	1.619	1.635
T2-O3	1.640(9)	1.632(4)	1.606(4)
T2-O4	1.671(8)	1.654(3)	1.624(4)
T2-O5	1.641(9)	1.614(4)	1.644(4)
T2-O6	1.633(13)	1.631(5)	1.620(5)
**<T2-O>**	1.646	1.633	1.623
**T-O-T angles (°)**			
T1-O1-T1	127.9(1)	134.7(1)	136.8(1)
T1-O2-T1	146.9(1)	152.8(1)	168.0(1)
T2-O3-T2	138.7(1)	140.5(1)	149.6(1)
T1-O4-T2	144.0(1)	147.6(1)	141.8(1)
T2-O5-T2	142.3(2)	141.6(1)	142.6(1)
T2-O6-T2	151.5(2)	154.2(1)	156.3(1)
**<T-O-T>**	141.8	145.2	149.2

KB-O3 [X6]	2.88(1)	KB-O3 [X6]	2.88(1)	KB-O3 [X6]	3.01(1)
KB-O5 [x6]	3.37(1)	KB-O5 [x6]	3.37(1)	KB-O5 [x6]	3.38(1)
KC-O4 [X8]	3.28(1)	KC-O4 [X8]	3.32(1)	KC-O4 [X8]	3.32(1)
KC-O5 [X4]	2.94(1)	KC-O5 [X4]	2.95(1)	KC-O5 [X4]	2.95(1)
KD-O4 [X4]	3.15(1)	KD-O4 [X4]	3.18(1)	KD-O4 [X4]	3.28(1)
KD-O6 [X2]	2.99(1)	KD-O6 [X2]	3.07(1)	KD-O6 [X2]	3.14(1)
KD-W2 [X2]	2.85(1)	KD-W1	1.32(2)	KD-W2 [X2]	3.12(1)
KD-W3 [X2]	3.64(1)	KD-W2 [X2]	2.88(1)	KD-W1	3.10(1)
W1-W2 [X2]	3.03(1)	KD-W3 [x2]	3.80(1)	K4-W3 [x12]	2.53(2)
W1-W3 [X2]	2.21(1)	W1-W2 [X2]	2.11(1)	K4-W6 [x2]	2.10(1)
W1-W3 [X4]	2.79(1)	W1-W3 [X2]	2.65(1)	W1-W3 [x2]	2.59(1)
W2-W3 [X4]	2.96(1)	W2-W3 [X4]	2.84(1)	W1-W1 [x2]	2.30(7)
W5-W3 [X12]	3.05(1)	W3-W3 [X2]	2.02(1)	W1-W6 [x6]	2.90(7)
W5-W4 [X6]	2.19(1)	W3-W4 [X2]	2.24(1)	W2-W2 [x2]	3.10(4)
W3-O1	3.22(1)	W3-W5 [x12]	3.01(1)	W2-W3 [x4]	3.05(2)
W4-O1	2.80(1)	W4-W4 [X2]	2.25(1)	W2-W6 [x2]	3.50(1)
		W4-W5 [x6]	2.25(1)	W3-W3 [X2]	2.24(2)
		W3-O1	3.78(1)	W3-W3 [X2]	3.26(3)
		W4-O1	2.82(1)	W3-W6 [x6]	2.40(4)
		W4-O2 [x2]	3.54(1)	W6-W6	3.30(2)
				W3-O1	3.19(2)

Table S3 Adsorption kinetics obtained using 0.200 g of zeolite LTL and 1 mL of thallium solution with an initial concentration of 485 ppm. C_e = equilibrium concentration, q_e = adsorption capacity at equilibrium, Re = removal efficiency.

Material mass (g)	Solution volume (mL)	Contact time (min)	C_e (ppm)	q_e (mg g^{-1})	R_e%
0.202	1	5	1.03±0.33	2.39±0.03	99.79±0.07
0.200	1	10	0.77±0.02	2.42±0.01	99.84±0.01
0.201	1	30	0.96±0.44	2.40±0.01	99.80±0.09
0.203	1	60	1.23±0.51	2.39±0.02	99.75±0.11
0.203	1	120	1.22±0.51	2.39±0.01	99.75±0.10
0.202	1	1440	0.84±0.06	2.40±0.02	99.83±0.01

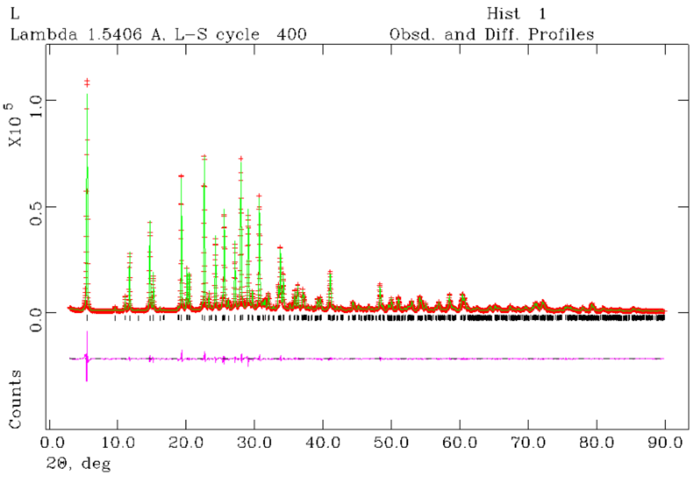

Figure S1 Observed and calculated diffraction patterns and final difference curve from Rietveld refinement of K-LTL.

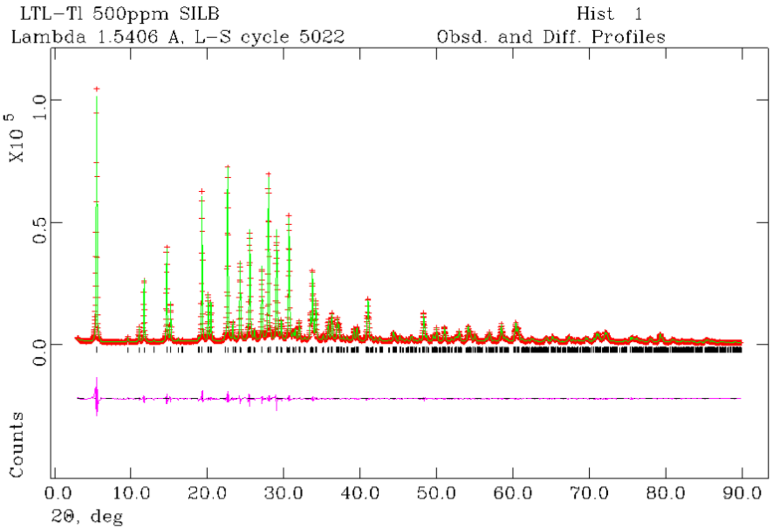

Figure S2 Observed and calculated diffraction patterns and final difference curve from Rietveld refinement of K-LTL Tl low.

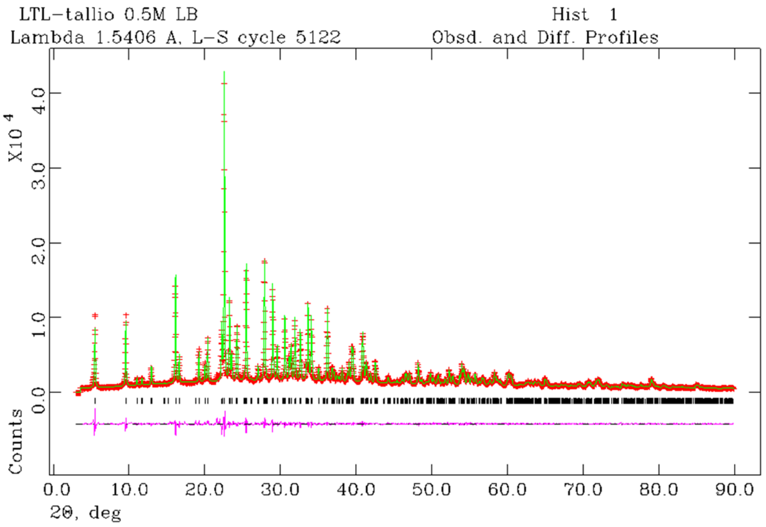

Figure S3 Observed and calculated diffraction patterns and final difference curve from Rietveld refinement of K-LTL Tl high.
